# Social determinants of alcohol and tobacco use among Hispanic adolescents: a scoping review

**DOI:** 10.3389/fpsyt.2025.1568462

**Published:** 2025-08-07

**Authors:** Kazi Priyanka Silmi, Victoria Castillo, Nallely Segura, Nayeli Carrillo Cervantes, Yailene Perez, Aubrey Valenzuela, Erika A. Pugh, Jennifer B. Unger, Marybel R. Gonzalez

**Affiliations:** ^1^ Department of Psychiatry and Behavioral Health, College of Medicine, Ohio State University, Columbus, OH, United States; ^2^ Department of Psychology, University of California San Diego, San Diego, CA, United States; ^3^ Department of Population and Public Health Sciences, University of Southern California, Los Angeles, CA, United States

**Keywords:** Hispanic, youth, alcohol use, tobacco use, mental health, social determinants of health

## Abstract

**Background:**

Alcohol and tobacco use (ATU) have been persistent public health concerns among youth in the United States (U.S.), including Hispanic youth who represent 25% of all American youth. While the psychosocial factors associated with ATU among Hispanic adolescents have been investigated for decades, the social environments for youth have undergone considerable change over time. The aim of this scoping review was to examine how social determinants of health (SDOH) influence ATU among Hispanic adolescents and to assess the extent to which mental health variables are integrated into these studies.

**Methods:**

We conducted a systematic, reproducible search on PubMed, PsycInfo, and Scopus for empirical research articles published that examined the SDOH of ATU among Hispanic/Latino/a/x adolescents in the U.S. We retrieved and screened 1467 titles and abstracts that yielded 241 articles for full review, of which 63 articles met final criteria for inclusion in the final synthesis. We categorized the SDOH by domains of behavioral, physical/built environment, socio-cultural, health care, and by levels for individual, interpersonal, school, community, and societal factors.

**Results:**

Most studies focused on socio-cultural domains within individual and interpersonal levels. Less studies examined societal and healthcare domains. Along with the influence of individual and interpersonal determinants (e.g., generational status, Hispanic values, acculturation stress, family and peers), community level factors (e.g., neighborhood level factors such as neighborhood exposure to ATU and neighborhood ethnic concentration) emerged as key structural predictors of ATU. Ethnic discrimination was also identified as a societal level predictor that influenced ATU, with potential association of mental health as mediators, moderators, or co-occurring outcomes. About one-third of the studies investigated the influence of SDOH on ATU along with mental health related variables like depression, anxiety, and stress.

**Conclusion:**

SDOH at the societal level and in the health care domain were identified as understudied among Hispanic adolescents. Future research is needed on these broader societal and structural determinants, including access to healthcare services and the integration of substance use prevention within these services, to intervene early in adolescence and reduce ATU related health consequences among Hispanic adults in the U.S.

## 1 Introduction

Alcohol and tobacco are the most commonly used substances among adolescents in the United States (U.S.), with far-reaching implications for health, development, and well-being across the lifespan. Hispanic youth in the U.S. are more likely to initiate substance use before age 13 compared to their peers (1) but are less likely to initiate addiction medicine treatment (2). Hispanic youth are also the fastest-growing minoritized population in the U.S.; one in four adolescents identified as Hispanic in the latest census (3). They disproportionately experience adverse social determinants of health (SDOH) that may increase risk for alcohol and tobacco use (ATU) (4). However, Hispanic youth may also share cultural assets that may be protective factors (5, 6). Thus, it is important to understand how these complex cultural, social, and structural contexts may exacerbate the vulnerability of Hispanic adolescents to substance use.

SDOH—the modifiable socio-structural conditions in which individuals are born, grow, live, work, and age—are intricately connected to ATU across the life course (7, 8). The Healthy People framework, a national initiative designed to set evidence-based objectives for improving the health of all Americans, emphasizes the critical role of SDOH in shaping health outcomes, advocating for addressing systemic factors such as education, economic stability, neighborhood environments, and healthcare access to improve population well-being (8). Hispanic adolescents face significant resource gaps, including a higher poverty rate (34.3% vs. 22.3% nationally) (9), lower attainment of higher education (25% vs. 40% nationally) (10), and higher rates of being uninsured (17.7% vs. 8.6% in the general population) (11). Such disparities illustrate the structural disadvantages that Hispanic adolescents may face in health-promoting environments. Additional determinants, such as discrimination, acculturation stress and family dynamics can influence early initiation of ATU and later ATU related problems (12–14). However, the interplay between these determinants and their unique influence on ATU among Hispanic adolescents is complex.

Among youth aged 12 to 17, approximately 1.5 million (22.4%) Hispanic American youth reported lifetime alcohol use, 1.2 million (17.8%) reported drinking alcohol in the past year, and 223,000 (3.3%) met the criteria for alcohol use disorder (AUD) in the past year, according to the 2023 National Survey on Drug Use and Health (15). 2.33 million (8.4%) Hispanic American middle and high school students reported current (past 30 days) use of any tobacco product, according to the 2024 National Youth Tobacco Survey (16). Psychological distress, such as symptomatology of depression, anxiety, and stress, are intricately linked to both SDOH and substance use. Hispanic youth are also likely to experience higher SDOH-related stressors like discrimination, differential parent-child acculturation, and neighborhood safety, which can exacerbate psychological distress symptomology and wellbeing (17–19). On the other hand, structural stressors and psychological distress may drive these youth to use substances as a coping mechanism (12–14). Factors of psychological distress may be predictors or mediators through which SDOH have synergized protective or risk influence on ATU. Despite this, existing literature often examines measures of mental health and substance use separately, failing to capture their potential intersections driven by common SDOH (5, 15). For Hispanic adolescents, lack of integrated services for mental health and substance use may further jeopardize service utilization due to systemic barriers, cultural stigmas, and lack of family insurance (13, 14). Thus, it is essential to assess the extent to which existing literature investigating SDOH and ATU also examines mental health well-being, such as psychological distress, to better understand the underlying mechanisms and promote overall positive mental health behaviors in early adulthood.

Adolescence is a critical developmental period and ATU during this time has considerable implications across the life-course. ATU may disrupt brain maturation, cognitive functioning, and health risk behaviors that persist into adulthood. This subsequently increases the risk for SUDs and psychological distress (20). There is evidence that heavy ATU in adolescence adversely impacts cognition 10 years later, even when the individual is no longer using substances (21). While these outcomes may be exacerbated by factors such as socio-economic status (SES), family dynamics, and access to health-promoting resources among Hispanic adolescents (22), these risk factors are modifiable and show promise for intervention initiatives. Addressing these determinants through early preventive interventions during adolescence is essential to improve health outcomes across the lifespan.

This scoping review aims to address two critical questions: (i) What are the social determinants of ATU among Hispanic adolescents in the United States? and (ii) How are mental health-related variables incorporated in studies examining the associations between SDOH and ATU in this population? By mapping the existing literature, this review seeks to describe trends of commonly investigated factors and identify current knowledge gaps in the SDOH of ATU among Hispanic youth. In this review, we used the terms Hispanic to refer to studies that investigated ATU in adolescents who identified as Hispanic, Latino/a, Latinx, or with socio-cultural heritage from any Spanish-speaking U.S. Territory or Latin American region/country (e.g., Puerto Rican or Mexican American).

## 2 Methods

### 2.1 Study design

Utilizing Arksey & O’Malley’s framework for conducting a scoping review, we followed the five stages to design this study: research question formulation, relevant studies identification, study selection, data charting, and result reporting (23). We report our results using the Preferred Reporting Items for Systematic Reviews and Meta-Analyses extension for Scoping Reviews (PRISMA-ScR) guide (24). The protocol for this study was not registered or published. We utilized the Healthy People Framework in conjunction with the adolescent adaption of the National Institute on Minority Health and Health Disparities (NIMHD) framework to conceptualize and synthesize this review (25, 26).

### 2.2 Eligibility criteria

**Table 1** summarizes the criteria for inclusion and exclusion criteria of the articles. Inclusion and exclusion criteria consisted of the following:

- Peer-reviewed original research published in English: Only articles meeting this criterion were considered. No limits were placed on the publication date of the studies.
- Population in the U.S. states and territories: Studies conducted outside the United States were excluded, as the NIMHD framework was developed considering the historical and societal context of the United States. SDOH for ethnically minoritized groups may differ significantly in other countries.
- Adolescent age range (10–19): We used the adolescent age range 10-19, following the definition of the World Health Organization (27). Articles were also included if they did not explicitly mention the age range but reported grades in school corresponding to adolescents. Although 18–19-year-old college students were eligible, none of the screened studies reported disaggregated outcomes specifically for this group. For studies with samples including participants over 19 years old, we only included them if the mean age was within the adolescent range or if disaggregated results for the target age group were provided. Longitudinal studies that measured adolescent alcohol or tobacco use but reported outcomes only for participants older than 19 were excluded.
- Self-reported ethnicity as Hispanic or Latino/a/x, regardless of race: articles were included if they reported ATU of Hispanic or Latino/a/x adolescents. Studies reporting on samples containing multiple racial and ethnic groups were included if they reported disaggregated results for Hispanic or Latino/a/x adolescents or if there were specific findings on Hispanic youth in samples representative of more than 25% of the sample consisted of Hispanic youth.
- Predictor indicating SDOH: Only studies that explicitly included predictors related to social determinants of health were included. Studies that only reported associations between ATU outcomes and sociodemographic characteristics were excluded, as these constructs do not directly measure the modifiable social and environmental drivers directly contributing to health outcomes (28).
- Outcome of alcohol use, tobacco use, or combined alcohol and tobacco use: Eligible studies reported outcomes via adolescent self-report, parent-report, or laboratory tests. Articles conceptualizing “substance use” as an aggregate of alcohol, tobacco, and other substances (e.g., marijuana or illicit drugs) were excluded. Studies reporting only intentions or behaviors related to ATU (e.g., drunk driving, alcohol-induced risky sexual behavior, expectancy behaviors) were also ineligible. Additionally, studies with outcome variables reporting outcomes of ATU in adolescent age range were included; longitudinal studies that had adolescent measures but only reported ATU outcomes at an older age (>19) were excluded.

### 2.3 Search strategy and information sources

The search terms were constructed using a PECO (population, exposure, comparison, outcome) framework (29). The population was limited to U.S.-based Hispanic/Latino/a/x/identifying adolescents in the age range of 10-19. The exposures included variables encompassing the social determinants of health (SDOH) in the five domains of the Healthy People 2030 framework: economic stability, education access and quality, health care access and quality, neighborhood and built environment, and social and community context (8). The outcomes were empirical measures of ATU; although our inclusion criteria did not have limits of self-report or biochemical validation, the studies that met the inclusion criteria all represented adolescent self-reports.

We built a reproducible, systematic, and comprehensive search strategy through collaboration and consultation with the subject expert librarian at Ohio State University (detailed search terms are available in the **Supplementary Materials**). An initial search on PubMed identified the index terms, and then Medical Subject Heading (MeSH) terms were incorporated in the search. Search terms were iteratively refined for each database to retrieve articles from the following databases: PubMed, PsycInfo, and Scopus. The search strategies were built based on a May 15, 2024, search, and the latest one was conducted on June 10, 2024.

### 2.4 Data extraction and data synthesis

The retrieved list from the search results was pooled and uploaded to Covidence systematic review software for screening (30). At least two reviewers screened each title and abstract to ensure rigor, and any conflicts were resolved through discussion and consultation with senior author (MRG). Once the titles and abstracts were screened, a reviewer independently conducted the full-text evaluation following the inclusion and exclusion criteria. The team met weekly to discuss the requirements to ensure systematic inclusion and exclusion of articles.

**Figure 1:**
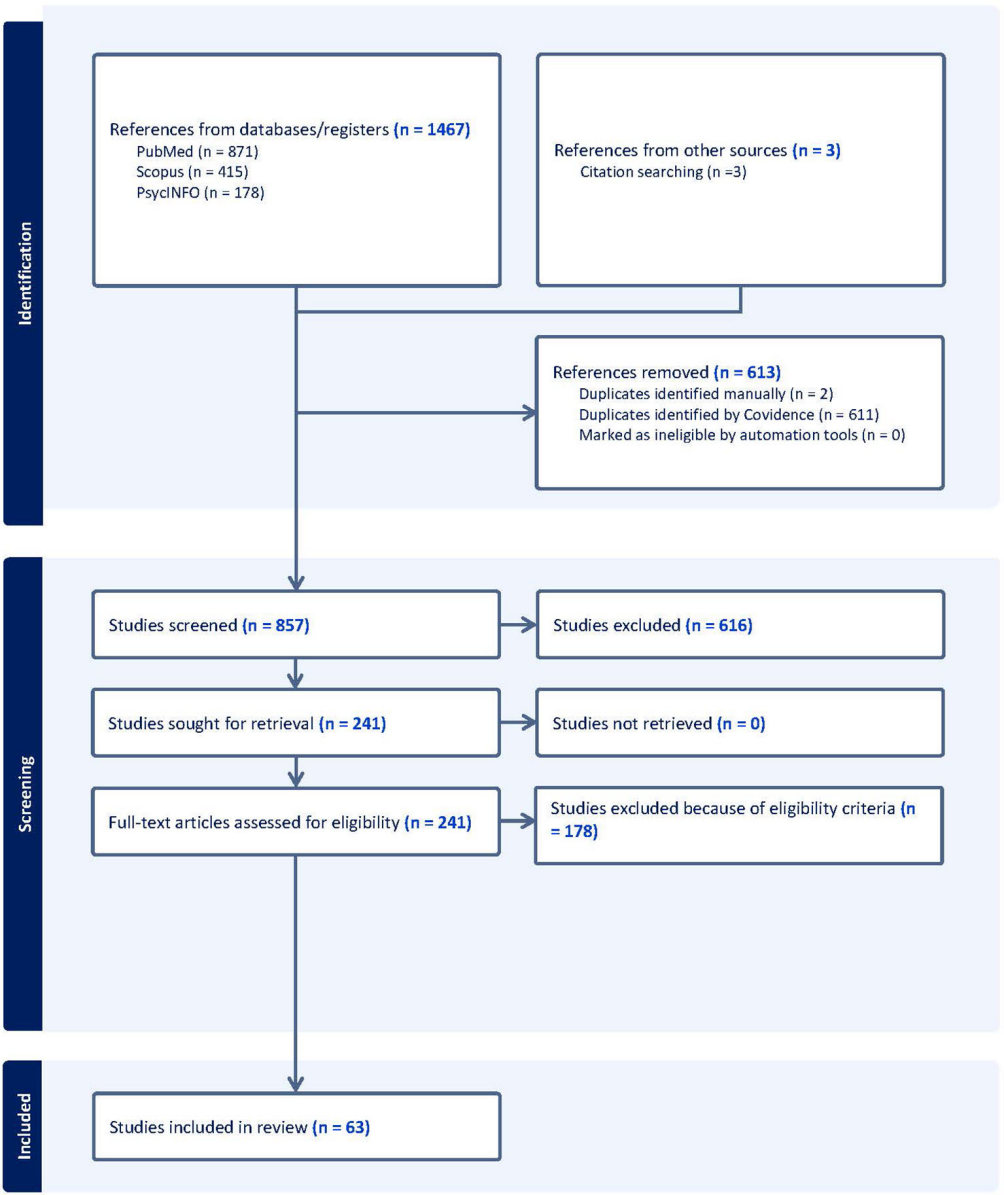
PRISMA flowchart.

The database search yielded 1467 potential peer-reviewed articles across all databases. After title and abstract screening for relevance, 241 studies remained for full text review among which 60 articles were included for final extraction. An additional 3 articles were identified through reference mining making the final count of articles synthesized to 63. **Figure 1** depicts the PRISMA flowchart describing the study selection process.

Data from the articles included in the final selection were extracted and charted for descriptive characteristics. Following that, we recorded the SDOH predictor variables that were reported to have significant association with ATU outcomes in each article. The significant predictor variables in the selected studies were categorized using the NIMHD framework adaptations for youth and mental health (25, 26). The NIMHD Research Framework is a multidimensional tool designed to investigate the complex and interconnected factors that influence health outcomes among minority populations. The original framework includes four levels of influences (individual, interpersonal, community, and societal); and five key domains of influence of health outcomes: biological (genetic, epigenetic, and physiological factors), behavioral (individual behaviors, preferences, and status), physical/built environment (environmental exposures, housing, neighborhood conditions, infrastructure), sociocultural environment (social norms, cultural values, discrimination, and acculturation), and healthcare system (access to care, quality of services, provider-patient interactions, and systemic biases). To adapt the framework for our adolescent population, we used the expanded model with five levels of influences that incorporated “school” as an additional level to account for the important impact of the school environment on adolescent outcomes (26). Additionally, we deliberately excluded the “biological” domain of influence to avoid reinforcing biological determinism, which could contribute to further stigmatization of minoritized youth (31). This decision aligns with our goal of investigating influences of social determinants of health, which we conceptualize as modifiable causes and social drivers of health.

**Table 1:** Criteria for inclusion and exclusion of articles.

Details	Inclusion	Exclusion
Type of Study	• Peer-reviewed original research • Analytical studies (case-control, cohort, cross-sectional)	• Reviews or meta-analysis • Protocol, commentaries, or editorials
Population	• Adolescents (10-19) living in the United States and territories • Identifying themselves as Hispanic, Latino/a/x, or with countries of origin in Spanish-speaking Latin America or Caribbean	• Outside the adolescent age range • Samples not in the United States
Main Outcome	• Alcohol Use • Tobacco Use • Substance Use (Alcohol and Tobacco Combined)	• Substance use measure aggregated with other substances (like cannabis or other drugs) along with alcohol and tobacco use • If alcohol and tobacco use outcomes specifically related to Hispanic/Latino/a/x adolescents were not reported in a disaggregated manner
Main Predictor	SDOH as defined by Healthy People 2030 Framework	• Not a SDOH as per Healthy People 2030 Framework • Studies that only reported differences by socio-cultural identities like race, gender, or sexual orientation

## 3 Results

### 3.1 Study characteristics

A total of 63 articles were included in the final synthesis. The study characteristics are summarized in **Table 2**. Of these, 57% (n=36) were cross-sectional studies and 43% (n=27) were cohort-based or longitudinal studies. 22% (n=14) utilized national surveys, while the rest were based on regional or community-based data. Among the included studies, 44% (n=28) articles exclusively focused on Hispanic/Latino/a/x youth, while the rest were included because their samples comprised mainly of Hispanic/Latino/a/x participants or reported disaggregated data specifically for Hispanic/Latino/a/x adolescents. Regarding substance use focus, 46% (n=29) examined alcohol use (AU), 22% (n=14) investigated tobacco use (TU), and 32% (n=20) explored alcohol and tobacco use (ATU) together.

**Table 2:** Study characteristics and corresponding significant Social Determinants of Health (SDOH) levels and domains of influences as per the NIMHD Framework.

Author	Outcomes	Type of study	Age range/Grade	Geographical region	Sample size	% of Hispanic/Latino/a/x in the full sample	Level of Influence of the predictor	Domain of Influence of the predictor
Acosta et al (32)	AU	cross-sectional	15-18	Miami, FL	371	100%	Individual, Interpersonal, Societal	Sociocultural Environment
Amin et al (33)	TU	longitudinal*	Age 12-21, Grade 7-12	U.S.- National	20,745	17.60%	Interpersonal, School	Behavioral, Physical/Built Environment, Sociocultural Environment
Arellan et al (34)	AU	cross-sectional	Grade 6-12	Southwestern U.S.	1805	67.31%	School	Sociocultural Environment
Assari et al. (35)	TU	cross-sectional	Age 12-17	U.S.- National	10619	22%	Interpersonal	Sociocultural Environment
Bacio et al (36)	AU	cross-sectional	Age 11-21, Grades 7-12	U.S.- National	2,482	100%	Interpersonal	Sociocultural Environment
Blount et al (37)	ATU	cross-sectional	Grade: Junior high	NYC	620	47.9	Community, Individual, Interpersonal, School	Physician/ Built Environment, Sociocultural Environment
Cano et al (38) (a)	ATU	longitudinal	14-17	Miami,FL; Los Angeles, CA	302	100%	Societal	Sociocultural Environment
Cano et al (39) (b)	AU	cross- sectional	Age 18-21	Not mentioned	129	100%	Community, Individual	Sociocultural Environment
Choukas-Bradley et al (40)	AU	longitudinal	Age 14-18, Grade 9-12	Not mentioned	364	21.70%	School	Sociocultural Environment
Dai et al (41)	ATU	cross-sectional	Grades 9-12/Age 14–18	U.S.- National	7705	25.40%	School	Sociocultural Environment
Eitle et al. (42)	AU	longitudinal	Grade 7-12	U.S.- National	7637	24.5	Interpersonal, School	Sociocultural Environment
Epperson et al (43)	TU	cross-sectional	Age 12–17	U.S.- National	12,474	25.4	Interpersonal	Sociocultural Environment
Epstein et al (44) (b)	AU	cross-sectional	Grade 7	New York City, NY	1410	100%	Community, Individual, Interpersonal, School	Behavioral Environment, Physical Environment, Sociocultural Environment
Flores et al (45)	AU	longitudinal	Age 12-15	Northern California	153	100%	Individual, Interpersonal	Health Care System, Sociocultural Environment
Forster et al (46)	ATU	cross-sectional	Grades 7-8	East Los Angeles, CA	184	80%	Individual, Interpersonal, School	Sociocultural Environment
Fraunglass et al (47)	ATU	cross-sectional	Grade 8	Little Havana area of Miami, FL	236	95%	Interpersonal	Sociocultural Environment
Goldbach et al (48)	AU	cross- sectional	Age 11-19	Los Angeles,CA; Miami, FL; El Paso, TX; Lawrence/Boston, MA	901	100%	Community, Interpersonal	Physical/ Built Environment, Sociocultural Environment
Gritz et al (49)	TU	longitudinal	Grades 5,8,12	Houston, TX	1441	20.60%	Individual, Interpersonal, School	Health Care System, Physical/ Built Environment, Sociocultural Environment
Guinn (50)	AU	cross-sectional	Age 9-12	Texas	937	100%	Individual, School	Sociocultural Environment
Huang et al (51)	AU	longitudinal	Age 11-14	U.S.- National	1080	15.65%	Individual, Interpersonal	Sociocultural Environment
Jackson et al (52)	AU	longitudinal	Age 12-17	Los Angeles, CA	889	59%	Community, Individual, Interpersonal	Physical/ Built Environment, Sociocultural Environment
Kandel et al (53)	TU	longitudinal	Grade: 7-12	U.S.- National	5374	18%	Individual	Behavioral Environment
Kreig et al (54)	AU	longitudinal	Age 12-17	U.S.- National	20,487	17.10 %	Community	Physical/ Built Environment, Sociocultural Environment
Kulis et al. (55)	ATU	cross-sectional	Grade 5	Phoenix, AZ	1374	100.00%	School, Societal	Sociocultural Environment
Lee et al (56)	ATU	cross-sectional	Age 12-17	U.S.- National	2621	100%	Individual, Interpersonal, School	Sociocultural Environment
Lee et al (13)	ATU	cross-sectional	12 or older	Denver, CO	736	52.71%	Community	Physical/Built Environment
Lorenzo-Blanco et al (57)	TU	longitudinal	Grades 9-11	Southern CA	1436	100%	Interpersonal	Sociocultural Environment
Lorenzo-Blanco et al (58)	ATU	longitudinal	14.51	Miami, FL; Los Angeles, CA	302(Los Angeles (N = 150) and Miami (N = 152)	100%	Interpersonal	Sociocultural Environment
Ma et al (59)	AU	cross-sectional	Age 13-16	U.S.-Southeast	225	100%	Interpersonal	Sociocultural Environment
Merianos et al (60)	AU	cross-sectional	US-national	U.S.-national	3457	100%	School	Sociocultural Environment
Morris et al (61)	TU	cross-sectional	Age 9-14, Grades 4-6	Pomona, CA	453	100%	Interpersonal	Physical/Built Environment, Sociocultural Environment
Nair et al (62)	AU	longitudinal	Grade 9-10	Suburban Atlanta, GA	252	100%	Interpersonal	Sociocultural Environment
Okamoto et al (63)	ATU	cross-sectional	Age 13-16, Grade 9	Los Angeles, CA	1332	73.30%	Societal	Sociocultural Environment
Osilla et al (64)	AU	cross-sectional	Age 14-18	Santa Barbara, CA	193	44.60%	Individual	Sociocultural Environment
Pandika et al (65)	TU	longitudinal	Grades 6-10, Grade 12	Rural Colorado; Kansas; Oregon; Utah; & Washington	3202*	60%	Interpersonal	Sociocultural Environment
Parker et al 1998(66)	TU	cross-sectional	Age 11-14, Junior High School	Southern California	545	56%	Community	Sociocultural Environment
Parra-Medina et al (67)	AU	longitudinal	Grade 7-9	San Diego, CA	554	100%	Individual, School	Behavioral Environment, Sociocultural Environment
Rew et al (68)	ATU	longitudinal	Grades 4-6	Texas	1,934	51%	Individual, School	Sociocultural Environment
Robles (69)	ATU	longitudinal	Grades 8-12	Puerto Rico	18,712	100%	Community, Interpersonal, School	Sociocultural Environment
Schwartz et al (70)	ATU	longitudinal	14-17, Grade 0	Miami,FL; Los Angeles, CA	302	100%	Interpersonal, Societal	Sociocultural Environment
Schwartz et al (71)	ATU	longitudinal	14-17, Grade 0	Miami,FL; Los Angeles, CA	302	100%	Community, Individual	Behavioral Environment, Sociocultural Environment
Shih et al (72)	ATU-(Together)	cross-sectional	Age 10-16, Grade 7-8	Southern CA	5550	59.00%	Individual, School	Behavioral Environment, Sociocultural Environment
Shih et al. (73)	AU	longitudinal	Age 10-16	Southern CA	6457	50%	Community	Physical/Built Environment
Song et al (74)	AU	longitudinal	Age 11-15, Grade 6-8	Central TX	602	100%	Individual, Societal	Behavioral Environment, Sociocultural Environment
Stanley et al (75)	AU	cross-sectional	Not reported	U.S.-contiguous	151,703	14.20%	Community, Individual	Physical/ Built Environment/ Sociocultural Environment
Steele et al (76)	AU	cross-sectional	Age 9-12	Chicago, IL	1156	46.00%	Individual, Interpersonal	Physical/ Built Environment/ Sociocultural Environment
Stroup-Benham et al (77)	AU	cross-sectional	Age 12-18*	US - Southwestern, Florida, and New York City, NY	825	100%	Interpersonal	Sociocultural Environment
Swaim et al 2011 (78)	AU	cross-sectional	Age 12-14, Grade 7-12	U.S. - Northeast, West, South and Midwest	213,225	10.05%	Community	Physical/ Built Environment, Sociocultural Environment
Tauras et al (79)	TU	repeated cross-sectional (1991-2010)	Age 14,16,18; Grades 8, 10, 12	U.S.- National	15,000-19,000	Not reported	Community, Individual, Interpersonal, School, Societal	Sociocultural Environment
Tobler et al (80)	AU	longitudinal	Age 11-14, Grade 6-8	Chicago, IL	5,655	29%	Community, Interpersonal, School	Physical/ Built Environment, Sociocultural Environment
Tobler et al (81)	AU	longitudinal	Grade 6-8, 12	Chicago, IL	4027	41%	Community, Individual, Interpersonal	Behavioral Environment, Physical/ Built Environment, Sociocultural Environment
Trapl et al (82)	TU	cross-sectional	Grade 6-12	Cuyahoga, OH	1,337	5.2	Individual, Interpersonal, School	Behavioral Environment, Physical/ Built Environment, Sociocultural Environment
Unger et al (83)	ATU	longitudinal	Grades 9-11	Southern California	2969	92%	Individual, School, Societal	Sociocultural Environment
Valdez et al (84)	ATU	cross-sectional	Age 14-18	Not reported	445	100.00%	Community	Physical/ Built Environment
Vega et al (85)	AU	cross-sectional	Under 18*	California	825	100%	Interpersonal	Health Care System
Wagner et al (86)	ATU	cross-sectional	Grade 9	Southern California	255	83%	Interpersonal	Sociocultural Environment
Wahl et al (87)	AU	longitudinal	Grade 7-12	U.S.- National	7992	19.22%	Community, Individual, Interpersonal, School	Behavioral Environment, Sociocultural Environment
Wallace et al (88)	TU	longitudinal	Grade 8	U.S.- National	36,000	14% (7% Mexican American, 5% Other Latinas,2% Puerto Rican)	Interpersonal, Individual	Sociocultural Environment
West et al (89)	ATU-(Together)	cross-sectional	Age 13-19,	San Diego, CA	205	100%	Community, Individual, Interpersonal, School	Behavioral Environment, Physical/ Built Environment, Sociocultural Environment
Wilkinson et al (90)	TU	longitudinal	Age 11-13	Texas	1087	100%	Community, Individual, Interpersonal	Behavioral Environment, Health Care System, Sociocultural Environment
Yan et al (91)	AU	cross-sectional	Age 11-13	Montgomery County, MD	322	100%	Community, Individual, Interpersonal, School	Behavioral Environment, Sociocultural Environment
Yockey et al (92)	TU	cross-sectional	Age 12-17, Grade 7-12	Cincinnati, OH	4,602	100%	Community, School	Behavioral Environment, Sociocultural Environment
Zhen-Duan et al (93)	ATU	cross-sectional	Age 14-18	U.S.-National	2493	100%	Interpersonal, School	Sociocultural Environment

### 3.2 NIMHD framework: domains and levels of influence

**Figure 2:**
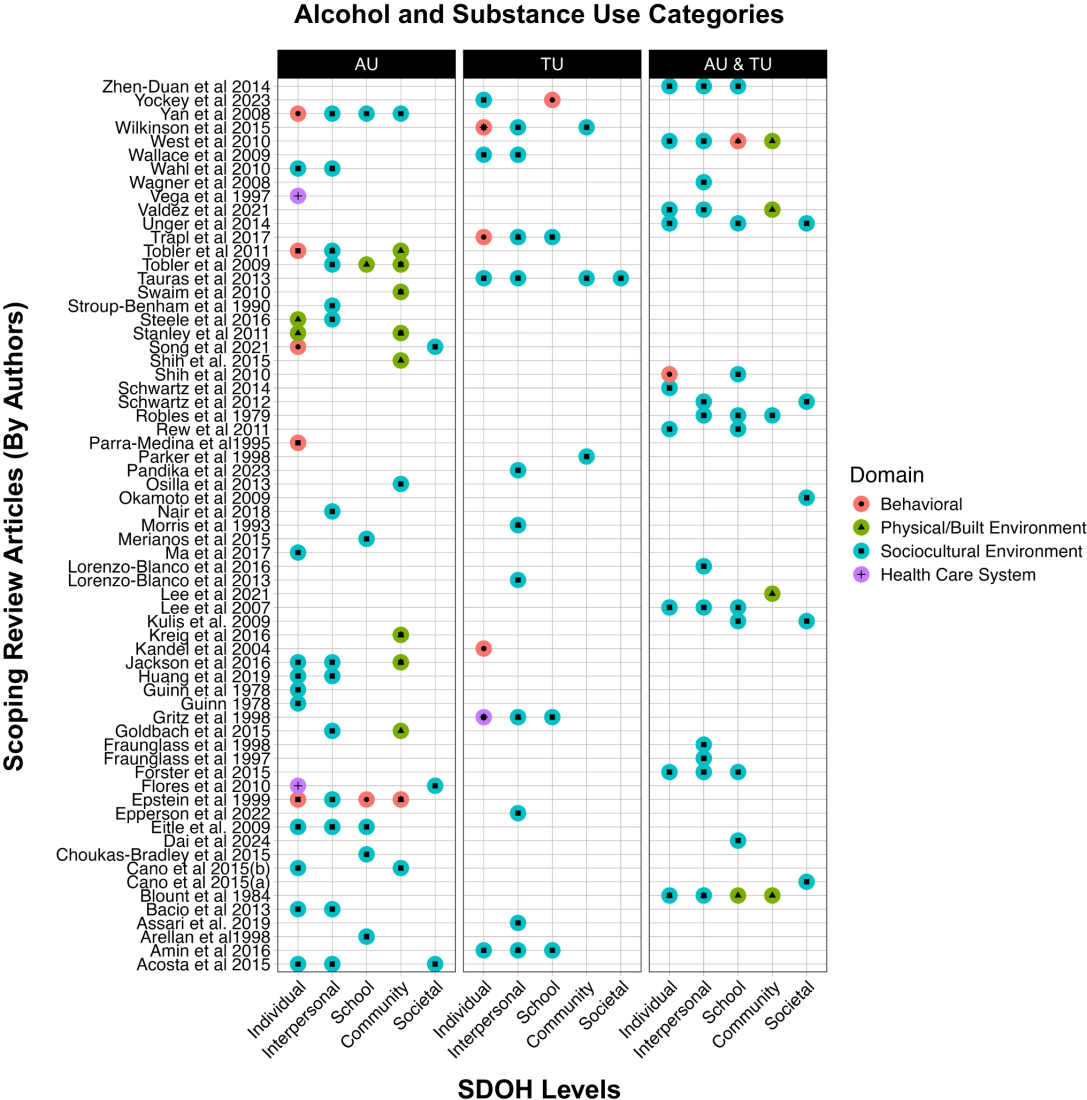
The plots displays all articles (y-axis) included in the scoping review and the corresponding I levels of SDOH (x-axis) that the studies found significant in association with alcohol use (AU), tobacco use (TU), or alcohol and/or tobacco use (ATU) combined. The levels of SDOH are color coded by the NIMHD Domains for Behavioral (red), Physical/Built Environment (Green), Sociocultural Environment (Blue), and Health Care System (purple).

Using the NIMHD framework, we categorized the SDOH predictors from the included studies by domains and levels of influence (**Figure 2**). Among the 348 SDOH measures identified in the included studies, 78% fell under the domain of sociocultural influence, emphasizing its critical role during the developmental stages of adolescence. The other domains of influence consisted of behavioral 9% (n=30), physical/built environment 12% (n=42), and healthcare system 0.14% (n=5). Notably, nearly all SDOH variables within the healthcare system domain were individual-level measures. As for levels of influence, 28.45% (n=99) variables were individual-level, 37.93% (n=132) were interpersonal level, 13.79% (n=48) were school level, 13.22% (n=46) were community level, and 6.61%(n=23) were societal level.

It must be noted that the levels and domains of measures in this study are indicators of peer-reviewed published articles that investigated influence of SDOH measures in their study, rather than published literature of all determinants of ATU of Hispanic adolescents. **Figure 2** summarizes studies across levels of influence in the NIMHD framework. Other results by specific outcome categories are available in the **Supplementary Materials**.

#### 3.2.1 Individual-level

Individual-level factors encompass youth characteristics, attitudes, and behaviors at the person-level that may influence risk of substance use. In this synthesis, individual level-factors assessed included: individual demographic characteristics, such as age, generational status, or country of origin, physical and mental health conditions, attitudes or beliefs toward substances, and individual practices/cultural beliefs.

Similar to the general adolescent population in the U.S. (24, 25), older age or progression to a more advanced stage of adolescence was consistently reported as a significant risk factor for ATU for Hispanic youth (44, 51, 52, 56, 87, 94). In the Hispanic population, generational status has been a significant predictor for alcohol use as well as tobacco use. First-Generation Immigrants tend to have lower rates of ATU compared to their U.S.-born counterparts (26, 52, 56, 87), a phenomena termed the “immigrant paradox” (36), in which immigrants exhibit better health outcomes despite socio-economic challenges. Substance use rates often increase with subsequent generations (36).

Since second and higher generational status indicated higher acculturation, studies also investigated acculturation through multi-level indicators spanning across different levels. At the individual level, the influence of the sociocultural indicators of acculturation were somewhat mixed. Whereas some Hispanic cultural values, like *simpatía* (valuing interpersonal harmony) may have some protective effect for alcohol use, others like familism (valuing family connection), *respeto* (valuing respect towards authority), and ethnic pride did not have a significant association with alcohol use (51, 94). Ethnic identification (identifying with one’s heritage) arose as both risk and protective factors for alcohol use across different studies (32, 70, 71). When comprehensively studied with different acculturation dimensions like values, identification, and practices, Schwartz and colleagues (71) found ethnic identification to be a risk factor for alcohol use in both male and female Hispanic adolescents; while US identity, collectivist values, as well as individualistic values were found to be protective factors for binge-alcohol use (71). The study also found differences by gender: adolescent boys with Hispanic practices were significantly at higher risk of alcohol use than girls. On the other hand, collectivist values were a significant risk factor for tobacco use in boys and a significant protective factor in girls. This suggests that at the individual level, cultural factors may have gendered differences with ATU. Other interpersonal, community, and societal level acculturation factors (e.g., parent-adolescent differential acculturation, practices, values, discrimination, perceived context) are discussed in the corresponding sections.

Academic performance and career goals are often conceptualized as individual level factors. Academic achievement, including higher grades, was associated with lower ATU consistently across all studies (46, 47, 61, 80, 91). A recent study on e-cigarette use among Hispanic adolescents found that students who did not consistently achieve good grades were more likely to report vaping in the past 30 days (92). Some researchers operationalized grade point average (GPA) as a measure of selective acculturation, indicating positive adaptation to academic environments (87). College aspirations also emerged as a protective factor against ATU (33).

#### 3.2.2 Interpersonal level

Interpersonal-level factors include the influence of family, peers, and other social interactions within an adolescent’s personal environment. Family characteristics encompass household structure, family income, family support, and family acculturation. Peer-level characteristics involve substance use among peers, peer attitudes toward substance use, peer acceptance, and peer support. Additionally, social interactions include interpersonal discrimination and the influence of social groups like friends and family.

The profound influence of family structure and relationships on adolescent substance use is evident in numerous studies. Household structure, parental income, parental education, and subjective social status are all associated with ATU use among Hispanic adolescents. For instance, living in single-parent household or with only siblings was consistently identified as a risk factor for substance use (33, 42, 44, 52, 59, 71, 79, 93). Some studies found that adolescents with parents of lower SES or education were at greater risk for ATU (59, 71, 88). However, contradictory findings also emerged, with higher parental SES associated with increased alcohol use (42, 79). To explain this phenomenon, studies suggest that the higher parent SES may indicate more disposable income to acquire ATU products (90). However, other studies suggested that Hispanic adolescents from higher-income families often lived in neighborhoods or attended schools with a higher proportion of White peers, where peer influences rather than parental income likely drove the increased alcohol use (73, 90).

Parental acculturation stress and functioning also played a significant role in adolescent ATU (36, 77, 86). Studies also explored the mechanisms between discrimination and acculturation stress which will be discussed in the subsequent section. Substance use among family members—such as fathers, mothers, siblings, or other household members—was also a prominent risk factor (29, 30, 32, 35, 84). Gender differences were observed between association of caregiver substance use and alcohol use: sibling substance use was significant only for boys (94) and maternal substance use only for girls (94). Interestingly, one study found that a family history of substance use might serve as a protective factor for certain outcomes (51), possibly due to heightened awareness of the consequences of substance use.

Parental support, monitoring, and communication significantly influenced substance use behaviors in Hispanic adolescents (26, 28, 36, 50, 51, 73, 80). Higher parental support generally correlated with lower ATU, although variations were noted based on region, gender, country of origin, and whether parents or adolescents reported on communication. In a study that reported disaggregated influences of parental influence on ATU in adolescents, it was found that parental emotional support was a significant protective factor for urban girls and rural boys, but a significant risk factor for urban boys (93). A study reporting comparison of binge-drinking outcomes of adolescents from Mexican, Cuban, and Puerto Rican origins found that communication with parents was a significant protective factor only for Cubans and communication between parents (intergenerational closure) was a significant risk factor for Mexicans (42). Another study found similar differences in the influences of parent-adolescent communication on ATU between adolescents in Miami and Los Angeles (70). The same study also observed that adolescent-reported communication was a significant predictor of alcohol use, while parent-reported communication was not, suggesting different mechanisms underlie these two perspectives. Acculturation gaps between parents and children—termed differential parent-child acculturation—were also identified as a risk factor for ATU (35, 36, 77). These gaps may lead to conflict and stress within the family, contributing to substance use behaviors.

Peer influence, encompassing peer substance use, and favorable attitudes of peers towards substance use, is a well-documented factor in adolescent substance use, with peer substance use consistently associated with higher ATU among Hispanic adolescents (30, 31, 48, 54, 55, 58, 62, 73, 80, 82, 84). However, certain nuances were observed. While friends’ use of alcohol and marijuana was a risk factor for both alcohol use as well as tobacco use, the effects varied. In some studies, peer substance use exerted a stronger influence on tobacco use compared to alcohol use (37). Other research indicated that peers’ use of other illicit substances, such as marijuana or cocaine, had a more pronounced impact on tobacco use than on alcohol use (54, 67). Interestingly, friends’ use of illicit drugs was associated with lower ATU in some cases (37). Peer-status or popularity ranking at school was identified as risk factors for alcohol use in Hispanic adolescents (40). These findings suggest that the desire for social integration may heighten susceptibility to peer influences, leading to increased alcohol use.

#### 3.2.3 School level

School contexts can shape adolescent behavior, as adolescents spend a considerable portion of their time in this environment. Various studies have investigated how adolescents interact with and respond to school-related characteristics, highlighting both protective and risk factors for ATU.

Positive school experiences, such as academic encouragement and support, were associated with lower substance use (50, 69, 80). School enjoyment and participation in extracurricular activities generally served as protective factors against substance use (47, 50, 80). However, some studies identified nuanced risks in specific subpopulations. For example, only for rural boys, school involvement was a risk factor for alcohol use in one study (95).

School difficulties—such as being expelled, suspended, or dropping out—were significantly associated with lifetime and current tobacco use (47, 49, 62) and alcohol use (30, 37, 67). Additionally, higher levels of school absenteeism were linked to greater lifetime ATU (30, 48). Detention and disciplinary actions, such as getting into trouble at school, were significant risk factors for both alcohol use as well as tobacco use. While dropping out of school was identified as a risk factor for alcohol use overall, a study reported that being male rather than being Hispanic, significantly moderated that association (34).

Access or exposure to substances within school settings posed a clear risk for ATU. Similarly, exposure to advertisements for alcohol and tobacco near schools was associated with increased substance use among adolescents (80). School-based ethnic discrimination was a significant risk factor (33) whereas school co-ethnicity (higher proportion of Hispanic which may indicate lower levels of school-based ethnic discrimination) was a protective factor for alcohol use (42). Discrimination at school can exacerbate stress and diminish school engagement and academic interest, contributing to increased ATU (41). However, studies exploring this specific mechanism at the school level are limited and warrant further investigation.

#### 3.2.4 Community level

Community-level factors encompass neighborhood and community characteristics that can be either risk or protective for ATU among Hispanic adolescents.

One of the earliest studies on neighborhood characteristics examined the intricate relationships between community environment and peer relationships in Hispanic adolescents (37). This study found that unsafe neighborhood characteristics were significantly associated with tobacco use but not alcohol use. Furthermore, these characteristics moderated the effects of interpersonal risk factors, such as participation in street culture and peer substance use, as well as individual-level protective factors like survival orientation.

More recent studies highlighted that neighborhood stressors, including gang violence, widespread drug availability, and social disorders serve as significant risk factors for tobacco initiation (13) and binge drinking (48). A study conducted in Hispanic adolescents residing along the U.S.-Mexico border reported that perceived disordered neighborhood stress was associated with current alcohol use but not tobacco use. Additionally, border community and immigration stress were significant predictors of elevated ATU (84).

Several studies also identified higher exposure to alcohol advertisements and a greater density of alcohol outlets as significant risk factors for alcohol initiation and current alcohol use among Hispanic adolescents (40, 94). Conversely, two studies reported that adolescents in Mexican American communities with greater physical availability of alcohol were less likely to report current alcohol use (75, 80). Further investigation revealed that higher alcohol availability in these neighborhoods was associated with lower access to alcohol at home, which served as a protective factor against adolescent alcohol use (80). Similarly, greater distance to alcohol outlets was linked to lower alcohol and tobacco initiation. However, this protective effect was mitigated when parental monitoring was inconsistent. This suggests that parents may reduce their vigilance if they perceive adolescents lack easy access to alcohol (89). Additionally, area deprivation—a measure of neighborhood disadvantage—was indirectly linked to alcohol use among 8th-grade Hispanic adolescents through mediators such as home alcohol access, deviant peer affiliations, and beliefs favorable to substance use (81).

Racial and ethnic composition within neighborhoods were also investigated as influential factors that may influence ATU in Hispanic adolescents. Neighborhoods with a higher concentration of Hispanic Americans and immigrants, or ethnic enclaves which may provide social support (42), were found to be protective against alcohol initiation, current alcohol use, and binge drinking among Hispanic adolescents (52, 54, 78). One study found that this protective effect of ethnic enclaves was attenuated when adolescents were exposed to diverse, non-residential neighborhoods (52). Living in integrated neighborhoods, where no single group represented more than 70%, also served as a protective factor (54). On the other hand, living in white communities was a significant risk factor of lifetime and recent alcohol use for Hispanic adolescents (78, 80).

Urban and rural community settings also influenced ATU. Hispanic adolescents in rural areas reported higher rates of alcohol initiation (78) and current tobacco use (79) compared to their urban counterparts. Rural community contexts often interact with interpersonal and school-level factors. For example, rural boys with greater school involvement and lower parental emotional involvement were at higher risk for current alcohol use, whereas these same factors had opposite effects in urban boys (95).

Studies specific to rural settings illuminated the SDOHs related to ATU among Hispanic adolescents. Research in rural communities explored factors such as tobacco access (65), family and peer influences (40), and environmental stressors (40, 60). While these findings contribute to understanding the rural context, studies on the ATU of Hispanic adolescents in rural areas remain limited in scope.

#### 3.2.5 Societal level

Societal-level factors encompass laws, policies, and social norms or practices that contribute to health outcomes. A subset of studies examined the impact of societal-level factors on ATU among Hispanic youth, focusing primarily on price policies and sociopolitical stressors (i.e., feeling unwelcome by the majority culture).

Ethnic discrimination emerged as a significant predictor of substance use, with Hispanic adolescents who experienced discrimination reporting higher rates of ATU (34, 41, 44, 70, 75, 81, 86). Among studies that investigated both alcohol use and tobacco use, most found that the impact of ethnic discrimination was greater on alcohol use than on tobacco use (34, 81). However, one study involving younger, elementary-aged Hispanic adolescents reported comparable effects of discrimination on both substances (55). A longitudinal study also highlighted that Hispanic male adolescents who experienced ethnic discrimination at school were at elevated risk for current ATU, with gender and race/ethnicity moderating this relationship (41).

Ethnic discrimination was frequently studied alongside other socio-cultural stressors, such as a negative context of reception, bicultural or acculturative stress, and domains of acculturation, including linguistic acculturation, cultural orientation, and cultural practices. Domains of acculturation were conceptualized as interpersonal-level factors, although societal influences undoubtedly shape these domains. For instance, one study employed a latent variable combining measures of ethnic discrimination, negative context of reception, and bicultural stress, finding that greater cultural stressors were associated with increased odds of cigarette smoking and binge drinking, with similar effects across these outcomes (39).

In contrast, Schwartz and colleagues (70) found that a negative context of reception (i.e., feeling unwelcome by the majority culture), rather than ethnic discrimination, was a stronger predictor of ATU among Hispanic adolescents. This study also identified variations in the influence of perceived context of reception based on location (Miami vs. Los Angeles), country of origin, and whether perceptions were reported by parents or adolescents. For example, in Miami, adolescent-reported negative context of reception increased the risk of cigarette use and drunkenness via adolescent-reported parent-child communication. In contrast, parent-reported negative context of reception served as a protective factor against binge drinking. Interestingly, parent-reported negative context of reception was a risk factor for adolescent alcohol use through its impact on parent-reported parent-adolescent communication. Several studies explored the mechanistic pathways by which discrimination influenced ATU, identifying mediators such as differential parent-child cultural characteristics, parental acculturative stress, and negative affect (74).

For the studies that met our inclusion criteria for SDOH and ATU in Hispanic adolescents, the extensive focus was on sociocultural norms and structures, with less focus on effects of policies, laws, and regulations on this population. One study reporting on the influence of tobacco price policies suggested that increases in the price of cigarettes can reduce consumption of cigarettes, with greater impact on Hispanic and Black adolescents compared to other ethnic groups and (79).

### 3.3 Intersection of mental health and ATU among Hispanic adolescents

Among the 63 ATU studies we reviewed, 34% (n=15) of studies concurrently investigated mental health well-being such as symptomatology for depression, anxiety, trauma/PTSD, and psychological stress among Hispanic adolescents. Notably, a majority of these studies (n=11) were published within the past decade, reflecting a recent growing interest in the intersection of mental health well-being and substance use in this population. Depression (n=9) was the most frequently mental health variable examined as an outcome alongside ATU (n=4) (77, 78, 90, 95), predictors of tobacco use (n=2) (54, 67), and as a covariate (n=2) (38, 75). Three studies further highlighted measures of psychological distress as outcomes—such as depression, negative affect, and PTSD—served as significant mediators in the association between discrimination and alcohol use among Hispanic adolescents (32, 41, 58). Symptoms of anxiety and depression were identified as risk factors for tobacco use (31, 87).

## 4 Discussion

This scoping review provides a broad overview of the social determinants of ATU for Hispanic adolescents as reported in the published peer-reviewed literature. Our findings, organized by levels of influence and domains within the NIMHD framework, revealed that most studies focused on sociocultural determinants at the individual and interpersonal levels. Comparatively fewer studies examined school, community, or societal-level factors, with the health care domain across all levels being particularly underrepresented. Approximately one-third of the studies incorporated mental health variables, often examined as mediators or co-occurring outcomes in the pathways linking social determinants to ATU. While cultural values and family dynamics are important, there is a critical gap in understanding how structural factors, such as sociopolitical climate, educational and economic opportunity, healthcare access, and policy, shape ATU risk in Hispanic adolescents. These synthesized findings underscore the need for multilevel, integrated approaches that consider both mental health and substance use within broader systems of inequality.

At the individual level, older age, later generational status, and cultural identity factors were associated with increased risk of ATU, though findings on cultural values such as familism, respeto, and ethnic pride were mixed (32, 36, 42, 59, 65, 70, 73, 87). Interpersonal-level influences, including lower socioeconomic status (SES), limited parental education, family structure, parent-adolescent communication, and peer characteristics were identified as significant predictors, with studies showing nuanced gender and regional patterns (36, 42, 51, 52, 61, 67, 70, 79, 87, 95). At the school level, protective factors included academic achievement and school involvement, while risk factors included absenteeism, suspensions, and exposure to ATU or related advertisements near school environments (30, 33, 41, 47, 48, 50, 67, 69, 80). Community-level influences such as neighborhood violence, substance availability, and immigration-related stress increased risk of ATU, whereas ethnic enclaves, integrated neighborhoods, and physical distance from alcohol outlets offered protective effects (37, 42, 52, 54, 75, 78, 80, 94). Finally, at the societal level, ethnic discrimination and negative sociopolitical contexts were associated with higher ATU, often mediated by psychological distress (34, 41, 44, 59, 70, 75, 78, 81, 86, 89). Although health care is an important SDOH in relation to ATU, our review found that thehealthcare domain remains understudied across all levels of the NIMHD framework (34, 41, 44, 70, 75, 81, 86). Health care domain factors, including health literacy of substance use related harm, routine early screening during pediatric care visit, access to youth-friendly services, referral services, school health policies etc. were investigated sparsely although high-quality treatment for substance use disorder play essential roles in primary, secondary, and tertiary prevention of ATU (96–98).

There are multilevel and multi-domain influences of SDOH that increase risk of ATU initiation, use and later ATU-related problems in early adulthood (45, 63). The focus on individual and interpersonal levels sociocultural influences is evident in this scoping review. Recent literature suggests socio-political climate, such as immigration policies, can influence mental health well-being and substance use among Hispanic adults and youth (97, 99–101). Future investigations should focus on identifying resilience factors that mitigate these adverse effects and on fostering advocacy efforts to influence policymakers. Studies also need to venture beyond individual and family level adversities, like lower SES and parental education, to investigate how lack of opportunities potentially drive these socially toxic environments of adolescents through chronic stressors in their living environment (102).

At the school level, factors such as academic engagement, school participation, and college aspirations were identified as protective, while dropping out, access to ATU, school-level consumption, advertisement near school increased risk. School-level factors can be dependent on the quality of school environment and school policies. For instance, school drug enforcement policies and mandatory substance use education can reduce substance use among youth (103, 104). However, it is unclear the extent substance use related school policies serve as protective factors for Hispanic adolescents, since they are disproportionately impacted by structural marginalization through punitive school discipline which can potentially increase their risk of drop-out, depression, and substance use (105). Future studies are needed to determine whether specific school substance use policy are more effective (i.e., harm reduction versus abstinence policies) as well as the role of help-seeking behavior among youth in school settings. School health policies can serve an important role of the healthcare domain to address substance use and mental health of minoritized students. Besides health education, schools can create mechanisms to become first responders for mental health and substance use screening, support, and referral before the problem becomes chronic (106).

At the community level, research on neighborhood level characteristics suggest that ethnic enclaves may offer protective benefits for Hispanic adolescents by fostering cultural continuity and social support, which reduce the risk of early alcohol initiation and binge drinking (28, 42, 60, 90). However, it must be noted that these environments may also reinforce structural isolation and limit access to diverse educational or economic opportunities, highlighting the need for place-based strategies that balance cultural cohesion with expanded opportunity structures (28, 29, 77, 88, 90, 95). Research suggests that tobacco and alcohol companies focus their advertising on specific populations through tailored marketing strategies, such as higher density of advertisements in Hispanic neighborhoods, culturally specific branding, and sponsorship of events associated with these communities, contributing to increases in tobacco use (43, 53). Tobacco use during adolescence can impact brain development and increase risk for substance use disorders (SUD) and other adverse health outcomes later in adulthood (107, 108). Our review of the literature suggested higher cigarette prices was a protective factor while neighborhood advertisements were a risk factor for ATU in the Hispanic population (37, 51). The digital environment further complicates this landscape. Additionally, e-cigarettes and social media are emerging issues that impact behaviors of all adolescents. Hispanic adolescents may experience mental health distress due to messaging in social media (43, 96), which may increase their likelihood to turn to ATU as a coping mechanism, when promoted as normalized strategies via peer-driven and commercial social medial content promoting substance use (43, 96). Particular attention should be given to the SDOH driving e-cigarette use among Hispanic adolescents, given rising rates of use. Investigations need to explore how these digital SDOH, combined with peer influence shape ATU trajectories in stressed youth, and how regulations might mitigate such exposure.

Societal level factors, such as discrimination based on race, gender, sexuality, and immigration status can lead to minority stress, manifesting in mental health symptoms such as depression, anxiety, and PTSD (15, 102, 109). Among youth receiving health care services for substance use, co-occurring mental health conditions are frequently reported (7).While it is encouraging that recent studies are increasingly examining the co-occurrence of mental health issues and substance use among Hispanic adolescents, there remains substantial work to be done in this area. Research highlights pathways through which discrimination based on race, gender, and sexuality can lead to minority stress, manifesting in mental health symptoms such as depression, anxiety, and PTSD. Adolescents often turn to substance use as a coping mechanism for these stressors (110). Among youth receiving healthcare services for substance use, co-occurring mental health conditions are frequently reported (15, 109). However, integrated interventions that simultaneously address both mental health and substance use remain scarce (111). Addressing mental health as part of an upstream prevention strategy could mitigate the likelihood of adolescents resorting to substance use as a coping mechanism (112). The finding that mental health serves as a significant mediator in the relationship between discrimination and alcohol use among Hispanic adolescents underscores the importance of culturally relevant interventions. Programs tailored to Hispanic youth should incorporate content that acknowledges stressors such as discrimination and differential acculturation, making them more relatable and impactful. Additionally, primary care and mental health providers should be equipped with training to address discrimination-related stressors during discussions on mental health and substance use, thereby empowering adolescents with healthier coping strategies. Supportive school health policies should also take an integrated approach to address mental health and substance use through primary prevention (like education and skill-building programs) and secondary prevention (like screening, referral, and youth-friendly school-based health centers).

SDOH for youth substance use in the health care domain include barriers to health care access; alcohol and tobacco education, screening and counselling are not part of the routine pediatric care visit; and alcohol and tobacco related consequences emerge much later in adulthood (63, 74, 83). As noted earlier, Hispanic youth are more likely to experience higher poverty rates, lower educational attainment, and limited health care access. These structural barriers make the healthcare domain an important yet underexplored factor in addressing ATU among this population. Key healthcare predictors, such as health literacy, access to developmentally appropriate substance use education, early screening for moderate ATU, and availability of culturally competent, youth friendly treatment for substance use disorder, can influence outcomes for youth navigating sociocultural stress and economic hardship. Future studies should investigate how these factors shape ATU trajectories in Hispanic and other minoritized youth. Additionally, equipping primary care and mental health providers with training to identify and respond to discrimination-related stressors can empower adolescents to adopt healthier coping strategies and build enhance patient-provider relationships. Integrating such efforts into supportive school health policies through primary prevention (like education and skill-building programs) and secondary prevention (like screening, referral, and youth-friendly school-based health centers) is necessary.

From a research standpoint, many studies on ATU among minoritized adolescents focus on racial and ethnic differences using large-scale surveys (37, 47, 100–103). While these studies offer valuable insights, they often rely on race or ethnicity as proxies for social and environmental factors (28), and do not report on the influence of SDOH. Our study sought to understand the influence of SDOH focusing on studies that move beyond these comparisons to investigate the nuanced social determinants influencing ATU among Hispanic adolescents. Comparative research has examined alcohol initiation rates among Hispanic youth relative to other demographic groups, highlighting differences (113, 114); however, reporting prevalence rates alone is insufficient. It is critical to examine proximal social determinants that shape the lived experiences of Hispanic youth to identify actionable intervention points and address root causes of substance use. This shift from descriptive statistics to exploring mechanisms and contexts driving ATU among Hispanic youth can inform preventive public health strategies, healthcare services, and policies.

The NIMHD Research Framework serves as a valuable tool for SDOH by identifying specific influences and domains that can become focal points for policy change and intervention design. However, our study highlighted challenges in categorizing some proxy factors due to their multilevel influences. For instance, parental income, education, and marital status were categorized as individual-level factors, as they are often used as proxies for SES. However, these factors can also indirectly influence parental stress, which is closely associated with parental monitoring and social support. Similarly, health risk factors such as depression, anxiety, and obesity were classified as individual-level factors. Yet, these conditions may reflect structural determinants like the social environment or access to healthcare, which play a critical role in promoting well-being and addressing untreated health conditions. We call for researchers to investigate underlying SDOH factors that may drive these individual-level determinants. Whereas certain factors—such as age and country of origin—are non-modifiable, and others —like acculturation, identification with ethnic practices, or attitudes toward substances—can indicate personal preferences, these factors are shaped by complex sociocultural mechanisms that are indeed modifiable. By understanding these mechanisms, researchers and policymakers can develop targeted strategies to address root causes and promote positive health outcomes.

The differential influence of SDOH predictors on Hispanic adolescents by region, sex, generational status, gender, and sexual orientation was a key finding of the study. This highlights the fact that Hispanic adolescents are not a monolithic group, and their socio-cultural practices vary by country of heritage, generational status, and regionality. Future reviews investigating differences in SDOH predictors of ATU between Hispanic youth in the US versus Latin America and also including Latin America and Spanish-language literature may reveal distinct SDOH patterns due to differing policy/cultural contexts (115, 116). Future interventions need to consider unique aspects of the lived experience of Hispanic adolescents and adapt already existing interventions to reflect the specific needs of the population to be served. There have been a number of intervention efforts to target substance use of Hispanic youth targeting individual and interpersonal levels of influences like Familias Unidas (117), Unidos Se Puede! (118), Sembrando Salud (119), Bridges/Puentes (120), Strategic Structural Systems Engagement (SSSE) (108, 108), Nuestros Familia (121), Familias Preparando la Nueva Generación (FPNG) (122), Alcohol Treatment Targeting Adolescents in Need (ATTAIN) (123). These interventions primarily focused on increasing adolescent self-efficacy to avoid alcohol use and parental monitoring/communication. However, structural level interventions, such as barriers to access, social norm changing campaigns, and policies, can have wider and greater impacts on SDOH that affects health and wellbeing of adolescents (8, 25, 74). Thus, future research should focus on structural-level components and their interactions, adopting an integrated approach to addressing mental health and substance use among Hispanic adolescents.

## 5 Limitations, strengths and future recommendations

The findings of this study need to be contextualized considering several limitations. SDOH is a complex phenomenon that can consist of factors beyond the categorizations outlined in this manuscript. Thus, this review may have excluded studies that did not correspond to our criteria of SDOH yet may emerge as important determinants of youth substance use. Notably, this review may have missed important factors related to digital environment, since these are not conceptualized as SDOH in traditional frameworks, although these are important sources of ATU-related exposures at the interpersonal and social media community level. Future studies should investigate how internet and social media may act as a SDOH itself (124) or may directly affect other SDOH to serve as risk or protective factors of ATU and mental health among Hispanic adolescents. While our scoping review focused on traditional SDOH domains, the digital environment, particularly online discrimination (e.g., cyberbullying, racialized algorithms), social media exposure, and digital substance marketing, likely interacts with sociocultural and structural determinants to shape ATU among Hispanic youth. Future SDOH frameworks should explicitly incorporate digital determinants, like social media (124) as these spaces may amplify stressors (e.g., discrimination) or introduce new risks (e.g., targeted alcohol/tobacco ads). Research should explore how digital environments mediate or modify known SDOH-ATU pathways, especially for mental health.

The categorization of variables by domains and influences of the NIMHD framework may not be generalizable with other studies since there is no specific criteria for categorization. For example, another review that used the NIMHD framework categorized race-ethnicity in the biological domain (125), however, race-ethnicity are socially constructed constructs and should not be used as a proxy to biological differences (28). Since we used the scoping review design to focus on breadth over depth, this paper provides a broad overview to answer our research question and does not include risk of bias assessment and heterogeneity of results by measurement tools and study designs of the included studies. Since these studies used a variety of different designs and measures, a meta-analysis was not feasible. We did notice how different measurements of SDOH measures like discrimination can influence outcomes; future studies can focus on specific SDOH measures like discrimination, neighborhood characteristics, or SES to critically assess how the levels of measurement tool may influence significance of association with outcome. Additionally, we merged studies with search terms for Hispanic vs. Latino/a/x labels, and thus we are not able to appreciate the unique identities within each Hispanic/Latino/a/x sub-group (126). This may have obscured the nuanced understanding of ATU outcomes in this population that experiences differential socio-cultural, economic, and political realities and thus future studies should consider this heterogeneity of experiences among the Hispanic population.

However, our focus on SDOH-related influence of ATU in Hispanic adolescents emphasizes the need for interventions and policies that address multi-level determinants rather than solely targeting individual choices or cultural practices. We report disaggregated evidence specifically focused on Hispanic adolescents, a population whose unique needs may be missed due to aggregation with other groups—such as the broader Hispanic community being combined with other minoritized racial/ethnic groups, or adolescents being grouped with young adults under the general category of “youth” (127). The use of the multi-level, multi-domain NIMHD framework helped us to articulate a nuanced analysis of the structural and social determinants that went beyond the socio-ecological perspective.

## 6 Conclusions

This scoping review highlights that individual and interpersonal levels within the sociocultural domains are the most studied social determinants of ATU among Hispanic adolescents, while societal factors and health care domain remain under investigated. Although mental health potentially plays a key mediating role between societal-level SDOH like discrimination and ATU, there is limited attention to these socio-structural and policy-level factors. To get an in-depth understanding of mechanistic pathways to ATU, more studies need to focus on intersectional SDOH-related risk and protective factors representing multiple levels and domains to understand mechanisms that drive the developmental trajectories of Hispanic adolescents’ experience with substance use and mental health.
