# Supplementary material for: Social determinants of alcohol and tobacco use among Hispanic adolescents: a scoping review
Source: Front Psychiatry. 2025 Aug 7;16:1568462. doi: 10.3389/fpsyt.2025.1568462 (PMC12368362; doi:10.3389/fpsyt.2025.1568462)
Supplement: Supplementary file 1 [file DataSheet1.docx]

Supplementary Material

# Supplementary Data

Data Source: Search Strategies used in PubMed.

| Adolescent | ("Adolescent"[Mesh] OR "Adolescent" OR “Adolescents” OR “Adolescence” OR “Youth” OR “Youths” OR “Teens” OR “Teen” OR “Teenagers” OR “Teenager” OR "Minors"[Mesh] OR "Minors" OR “Minor” OR "Child"[Mesh] OR "Child" OR “Children”) |
| --- | --- |
| Latinx | ("Hispanic or Latino"[Mesh] OR "Hispanic or Latino" OR “Hispanic or Latinos” OR “Hispanic Americans” OR “Hispanic American” OR “Spanish Americans” OR “Spanish American” OR “Puerto Ricans” OR “Puerto Rican” OR “Hispanics” OR “Latinos” OR “Latino” OR “Latinas” OR “Latina” OR “Latinx” OR “Cuban” OR “Cubans” OR “Cuban Americans” OR “Cuban American” OR "Cuban people" [Supplementary Concept] OR "Cuban people" OR “US Latin American” OR "Mexican Americans"[Mesh] OR "Mexican Americans" OR “Mexican American” OR “Chicanos” OR “Chicano” OR “Chicanas” OR “Chicana” OR "Chicano people" [Supplementary Concept] OR “Mexican” OR “Mexicans” OR "Mexican people" [Supplementary Concept] OR "Mexican people" OR "Guatemalan people" [Supplementary Concept] OR "Guatemalan people" OR “Guatemalan” OR “Guatemalans” OR "Dominicans-Dominican Republic people" [Supplementary Concept] OR "Dominican people" [Supplementary Concept] OR "Dominican people" OR “Dominicans” OR “Dominican” OR “Salvadorian” OR "Central American People"[Mesh] OR "Central American People" OR “Central Americans” OR "Caribbean People"[Mesh] OR "Caribbean People" OR “Caribbean Peoples” OR “Caribbeans” OR "Caribbean Hispanic people" [Supplementary Concept] OR "Caribbean Hispanic people" OR "South American People"[Mesh] OR "South American People" OR “South American Peoples” OR “South Americans” OR “South American”) |
| Alcohol use | (("Alcohol Drinking"[Mesh] OR "Alcohol Drinking" OR "Alcohol Intake" OR "Alcohol Intakes" OR "Alcohol Drinking Habits" OR "Alcohol Drinking Habit" OR "Alcohol Consumption" OR "Binge Drinking"[Mesh] OR "Binge Drinking" OR "Binge Alcohol Consumption" OR "Underage Drinking"[Mesh] OR "Underage Drinking" OR "Adolescent Alcohol Drinking" OR "Adolescent Alcohol Intake" OR "Youth Alcohol Consumption" OR "Underage Alcohol Use" OR "Youth Drinking" OR "Adolescent Drinking" OR "Teen Drinking" OR "Teenage Drinking" OR "Adolescent Alcohol Use" OR "Adolescent Alcohol Consumption" OR "Underage Alcohol Consumption" OR "Legal Drinking Ages" OR "Minimum Drinking Age" OR "Minimum Drinking Ages" OR "Minimum Legal Drinking Age"))) |
| Tobacco use | ((“Tobacco Smoking"[Mesh Terms] OR “Tobacco Products"[Mesh Terms] OR “Smok*” OR “Cigarette*” OR “Tobacco” OR “Nicotine”)) |
| Social Determinants of Health | (((((((("Social Determinants of Health"[Mesh] OR "Social Determinants of Health" OR "Health Social Determinant" OR "Health Social Determinants" OR "Structural Determinants of Health" OR "Health Structural Determinants")) OR (("Poverty"[Mesh] OR "Poverty" OR "Extreme Poverty" OR "Poverty, Extreme" OR "Absolute Poverty" OR "Indigents" OR "Indigent" OR "Indigency" OR "Federal Poverty Threshold" OR "Low-Income Population" OR "Low-Income Populations" OR "Population, Low-Income" OR "Populations, Low-Income" OR "Low Income Population" OR "Low Income Populations" OR "Population, Low Income" OR "Populations, Low Income"))) OR (("Socioeconomic Factors"[Mesh] OR "Socioeconomic Factors" OR "Factor, Socioeconomic" OR "Socioeconomic Factor" OR "Factors, Socioeconomic" OR "Standard of Living" OR "Living Standard" OR "Living Standards" OR "Social Inequality" OR "Inequalities, Social" OR "Inequality, Social" OR "Social Inequalities" OR "High-Income Population" OR "High Income Population" OR "High-Income Populations" OR "Population, High-Income"))) OR (("Resource Limited Areas" OR "Resource Limited Area" OR "Resource-Limited Areas" OR "Resource-Limited Area"))) OR (("Rural Population"[Mesh] OR "Rural Population" OR "Population, Rural" OR "Populations, Rural" OR "Rural Populations" OR "Rural Spatial Distribution" OR "Rural Communities" OR "Communities, Rural" OR "Community, Rural" OR "Rural Community"))) OR (("Neighborhood" OR "Neighborhoods" OR "Neighborhood Characteristics"[Mesh] OR "Neighborhood Characteristics" OR "Neighborhood Disadvantage" OR "Neighborhood Disadvantages" OR "Neighborhood Safety" OR "Neighborhood Effects" OR "Neighborhood Effect" OR "Neighborhood Environment" OR "Neighborhood Environments"))) OR (("Racism"[Mesh] OR "Racism" OR "Everyday Racism" OR "Racial Discrimination" OR "Racial Discriminations" OR "Racial Bias" OR "Racial Prejudice" OR "Racial Prejudices" OR "Covert Racism" OR "Systemic Racism"[Mesh] OR "Systemic Racism" OR "Systematic Racism" OR "Structural Racism" OR "Structural Racisms" OR "Institutionalized Racism" OR "Institutional Racism" OR "Social Discrimination"[Mesh] OR "Social Discrimination" OR "Housing Discrimination" OR "Discriminatory Practices" OR "Discriminatory Practice" OR "Exposure to Discrimination" OR "Exposure to Discriminations" OR "Discrimination Exposure" OR "Discrimination Exposures" OR "Perceived Discrimination"[Mesh] OR "Perceived Discrimination" OR "Health Status Disparities"[Mesh] OR "Health Status Disparities" OR "Health Status Disparity" OR "Socioeconomic Disparities in Health"[Mesh] OR "Socioeconomic Disparities in Health" OR "Socioeconomic Disadvantage" OR "Socioeconomic Disadvantages" OR "Social Disparities in Health" OR "Social Disparity in Health" OR "Economic Disadvantage in Health" OR "Socioeconomic Disparities" OR "Socioeconomic Disparity" OR "Economic Disparities in Health" OR "Economic Disparity in Health" OR "Healthcare Disparities"[Mesh] OR "Healthcare Disparities" OR "Healthcare Inequalities" OR "Healthcare Inequality" OR "Health Care Inequalities" OR "Health Care Inequality" OR "Health Care Disparities" OR "Health Care Disparity" OR "Healthcare Disparity"))) |

# Supplementary Figures and Tables

## Supplementary Figure


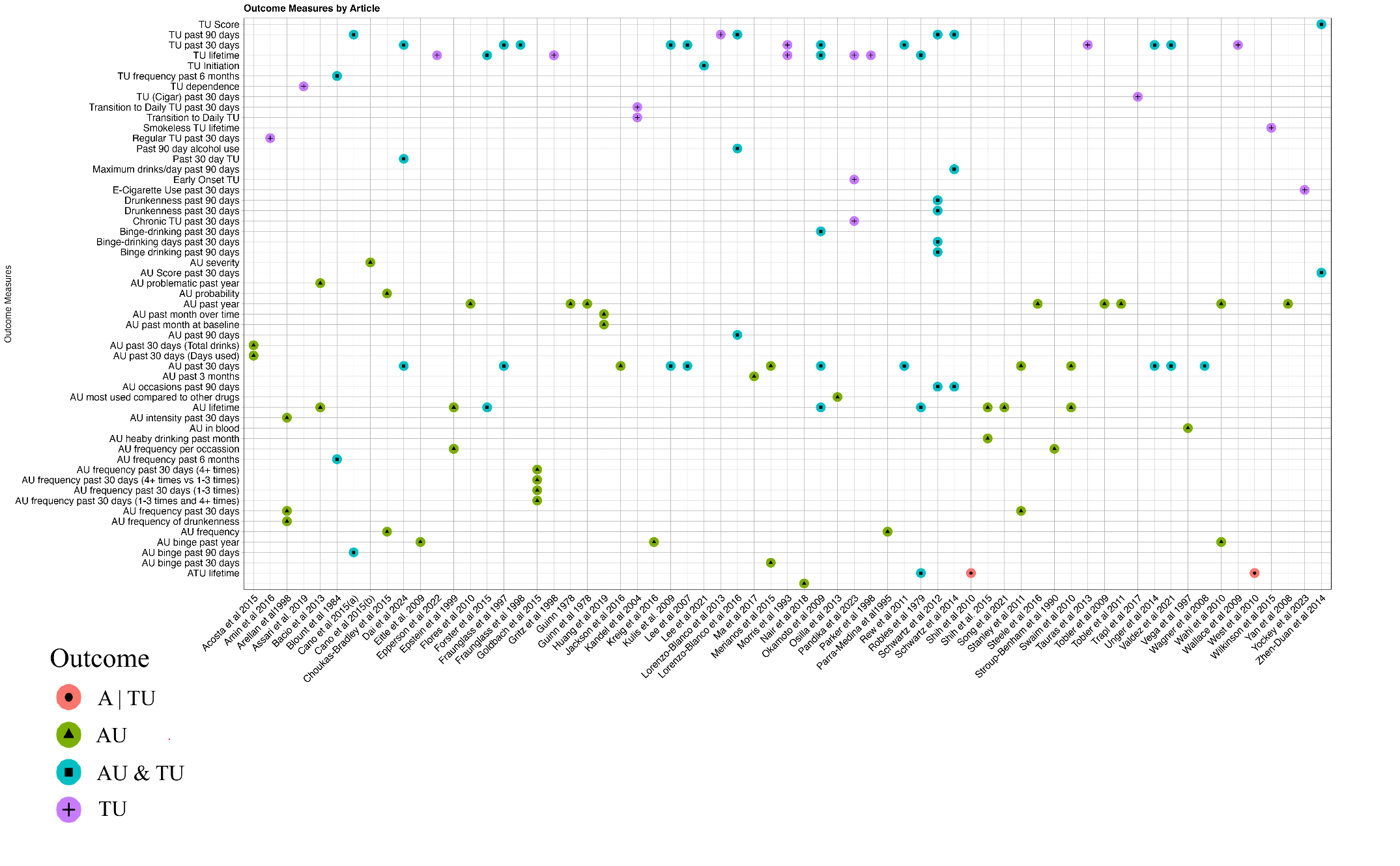
**Supplementary Figure** 1**. Articles summarized by Outcome measures (AU= Alcohol Use, TU=Tobacco Use, A|T U= Composite Measure for Alcohol or Tobacco Use, AU & TU= Disaggregate measure of Alcohol Use and Tobacco Use).**
